# Outbreak of *Salmonella* Enteritidis linked to the consumption of frozen beefburgers received from a food bank and originating from Poland: northern France, December 2014 to April 2015

**DOI:** 10.2807/1560-7917.ES.2016.21.40.30363

**Published:** 2016-10-06

**Authors:** Gabrielle Jones, Nathalie Pihier, Caroline Vanbockstael, Simon Le Hello, Sabrina Cadel Six, Nelly Fournet, Nathalie Jourdan-da Silva

**Affiliations:** 1 Santé publique France (the French public health agency), Picardy regional office, Amiens, France; 2 French General Directorate for Food, Paris, France; 3 Institut Pasteur, French National Reference Centre for *Escherichia coli, Shigella* and *Salmonella*, Paris, France; 4 Université PARIS-EST, French Agency for Food, Environmental and Occupational Health and Safety, Laboratory for Food Safety, Maisons Alfort, France; 5 Santé publique France (the French public health agency), Saint-Maurice, France

**Keywords:** Salmonella, food-borne infections, Outbreaks, epidemiology

## Abstract

A prolonged outbreak of *Salmonella enterica* serotype Enteritidis occurred in northern France between December 2014 and April 2015. Epidemiological investigations following the initial notification on 30 December 2014 of five cases of salmonellosis (two confirmed *S.* Enteritidis) in young children residing in the Somme department revealed that all cases frequented the same food bank A. Further epidemiological, microbiological and food trace-back investigations indicated frozen beefburgers as the source of the outbreak and the suspected lot originating from Poland was recalled on 22 January 2015. On 2 March 2015 a second notification of *S.* Enteritidis cases in the Somme reinitiated investigations that confirmed a link with food bank A and with consumption of frozen beefburgers from the same Polish producer. In the face of a possible persistent source of contamination, all frozen beefburgers distributed by food bank A and from the same origin were blocked on 3 March 2015. Microbiological analyses confirmed contamination by *S.* Enteritidis of frozen beefburgers from a second lot remaining in cases’ homes. A second recall was initiated on 6 March 2015 and all frozen beefburgers from the Polish producer remain blocked after analyses identified additional contaminated lots over several months of production.

## Introduction

While targeted control measures implemented in food production in France in the 1990s, notably in poultry, cattle and milk production, were shown to reduce the number of human *Salmonella* cases, *Salmonella* remains an important source of food-borne outbreaks in France [1]. In France, notification of food-borne disease outbreaks (FBDO) to the regional health agency (ARS) is mandatory for health professionals, clinical microbiologists and institutional catering services. Notifications can be made by telephone, email or fax using a standardised notification form. The ARS is responsible for case investigations while the Departmental Direction for the Protection of Populations (DDPP) is responsible for food safety investigations. The ARS then transmits the results of the outbreak investigations to Santé publique France (SpFrance), the French public health agency, which is responsible for epidemiological surveillance of FBDOs. In 2012, the notification rate for confirmed *Salmonella* cases in France was 13.3 per 100 000 population [2]. 

In addition to mandatory notification of FBDOs, private and hospital laboratories send *Salmonella* isolates on a voluntary basis to the dedicated French National Reference Centre (NRC) for serotyping and microbiological surveillance. In 2014, of 9,077 isolates received and analysed at the NRC, *Salmonella enterica* serotype Enteritidis (*S.* Enteritidis) was the third most frequently isolated serotype behind *S.* Typhimurium and monophasic variant 1,4,[5],12:i:- [3]. The same year, 1,380 food-borne outbreaks were notified to SpFrance, with *Salmonella* spp. representing the most common source in outbreaks with confirmed aetiology (110 of 254 FBDO (43%)) [4]. A recent study of the community incidence of salmonellosis in France from 2008 to 2013 estimated an annual community incidence rate of 307 cases per 100 000 population and 4,305 hospitalisations annually [5].

### The event

In December 2014, the ARS in Picardy was notified by a hospital laboratory of five cases of salmonellosis in young children residing in the Somme department in a single week, of which two were confirmed *S.* Enteritidis and three were *Salmonella* spp. The number of cases was unusual for the laboratory, which typically observes one to two cases of *Salmonella* isolated in young children for the same time period. Preliminary investigations were initiated by the Santé publique France Picardy regional office using a standardised *Salmonella* questionnaire to identify food items and place of consumption or purchase. All cases, or a guardian for minors, were interviewed and a single common link with the consumption of food products from food bank A was identified. Further investigations were undertaken to identify the source of infection and implement appropriate control measures. Here we present the results from the prolonged outbreak of *S.* Enteritidis occurring from December 2014 to April 2015.

## Methods

### Outbreak investigations

We defined a confirmed case as a person residing in the Somme, Nord or Pas-de-Calais department with laboratory-confirmed infection of *S.* Enteritidis after week 51 2014. A probable case was a person residing in the Somme, Nord or Pas-de-Calais department presenting symptoms compatible with *Salmonella* infection (abdominal pain, diarrhoea, with or without recorded fever), and an epidemiological link to a confirmed case, after week 51 2014. A possible case was defined as a person residing in the Somme, Nord or Pas-de-Calais department presenting symptoms compatible with *Salmonella* infection (abdominal pain, diarrhoea, with or without recorded fever), but no laboratory confirmation, after week 51 2014. Human-to-human transmission was suspected for cases presenting symptoms > 7 days after incident cases.

Cases were identified from three sources: (i) an active search of cases from private laboratories in the cities where initial cases were identified (two cities in the Somme department) and identification of cases in the families of incident cases representing outbreak clusters; (ii) mandatory notification of food-borne disease outbreaks occurring in northern France; (iii) microbiological surveillance data from the NRC of all confirmed cases sent by laboratories in the department were cases had been notified since week 51 2014. In parallel, the NRC also verified that no other French departments had presented an unusual number of cases of *S.* Enteritidis for the same time period. Cases, or a guardian for minors, were asked about their food consumption in the week before symptom onset using a standardised *Salmonella* questionnaire administered by telephone between 30 December 2014 and 22 April 2015. Information was collected on at risk activities (travel abroad, contact with animals) as well as food items consumed and the place of purchase of all food items.

### Microbiological and food trace-back investigations

In France, human *Salmonella* isolates received at the NRC are analysed using the White-Kauffmann-Le Minor scheme for serotyping and standardised multilocus variable tandem repeat analysis (MLVA) for comparison of cases in the context of outbreak investigations [6,7]. Food isolates were analysed by the Laboratory for Food Safety of the French Agency for Food, Environmental and Occupational Health and Safety (ANSES) using serotyping by agglutination followed by MLVA for *S.* Enteritidis, which is common practice in the case of food-borne outbreak investigations [7].

When available, *Salmonella* spp. isolates from notified cases were sent to the NRC for serotyping. Available food samples were collected for microbiological testing by the Laboratory for Food Safety of the ANSES. Ten human and food sample strains each were analysed by MLVA to confirm the microbiological link.

Food trace-back investigations were conducted by the DDPP in the Picardy region and by the General Directorate for Food (DGAL) for national and international investigations.

### European investigations

European health authorities were notified about the outbreak and of the suspected source of contamination via the European Commission’s Early Warning and Response System (EWRS), the European Epidemic Intelligence Information System platform (EPIS) run by the European Centre for Disease Prevention and Control, and the European Commission’s Rapid Alert System for Food and Feed (RASFF).

## Results

### Epidemiological investigations

A total of 45 cases identified from notifications by hospitals, medical laboratories, mandatory notification of FBDOs, and the NRC listing, were interviewed regarding their food consumption. Twenty-three were confirmed *S.* Enteritidis, 17 were probable cases and five were possible cases. An additional 26 confirmed cases identified only from the NRC listing were not interviewed either because they could not be contacted (four cases) or because the information was available only after implementation of outbreak control measures which took place on 22 January and 3 March 2015 (22 cases). These cases were not contacted because they occurred up to several weeks before control measures and the aim of investigations at this stage of the outbreak was to identify cases occurring after control measures in order to verify their efficacy.

**Figure 1:**
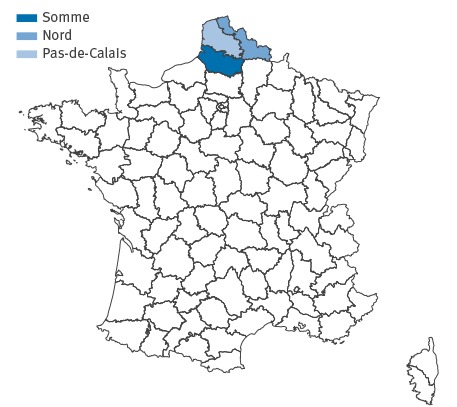
French departments of residence for identified confirmed, possible and probable cases of *Salmonella* enteritidis, northern France, December 2014 to April 2015

The first outbreak notification occurred on 30 December 2014 and the second on 2 March 2015. All 45 interviewed cases resided in adjacent departments in northern France: 37 cases in the Somme department, six cases in the Nord department and two cases in the Pas-de-Calais department (Figure 1). Information on age was known for 37 of 45 cases and ranged from 1 month to 49 years old (median age: 9 years). The male to female sex ratio was 1.1 (information available for 40 of 45 cases). In total eight children and one adult were hospitalised.

Of the 45 cases with food consumption information, 41 had consumed frozen beefburgers from food bank A and no other common source of infection was identified. Of the four remaining cases, three were human-to-human transmission with incident cases reporting consumption of frozen beefburgers from food bank A and one case reported no link with food bank A and no consumption of frozen beefburgers. In nine families (14 cases) specifying mode of cooking of the frozen beefburgers, only three (six cases) reported consuming the beefburgers well-done (no pink visible). A total of 11 FBDOs were identified, one by mandatory notification and ten during investigations of cases.

**Figure 2:**
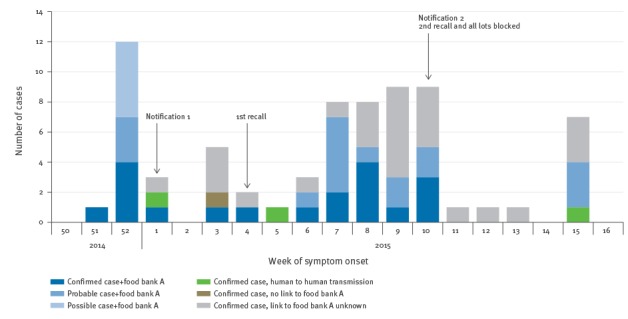
Possible, probable and confirmed cases of *Salmonella* Enteritidis, by week of symptom onset, northern France, December 2014 to April 2015 (n=71)

Symptom onset ranged from 21 December 2014 to 6 April 2015. The outbreak curve shows two outbreak waves (Figure 2). Four cases notified in week 15 2015 occurred in the same family, who reported regularly storing frozen beefburgers from food bank A for several weeks in their freezer.

For 28 cases with information on date of symptom onset missing, the date of strain isolation was used.

### Microbiological and food trace-back investigations

**Figure 3:**
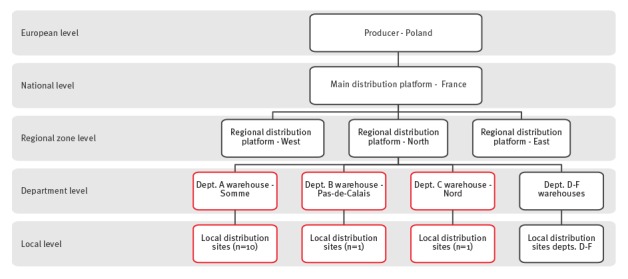
Schema showing the distribution network of frozen beefburgers originating in Poland and distributed by food bank A in northern France, December 2014 to April 2015

Following the initial notification and epidemiological investigations identifying a common link with food bank A, food trace-back investigations were initiated on 8 January 2015. The geographic distribution of the cases suggested a food source contaminated before distribution as cases frequented food bank A at different local distribution sites (10 sites in the Somme, one in the Nord and one in the Pas-de-Calais). While a total of six departments received frozen beefburgers from the suspected lot, cases were only identified in three departments (Figure 3). Visits to three local sites of food bank A as well as the regional distribution platform for northern France by the DDPP revealed no non-compliance in storage conditions or respect of the cold chain.

Dept: department.

Red boxes show the warehouses and local distribution sites at department level where the lots of beefburgers linked to confirmed, probable or possible *Salmonella* Enteritidis cases were identified.

As of 9 January 2015, food bank A temporarily blocked the distribution of all lots of frozen beefburgers. Based on the dates and locations of food bank A sites frequented by the cases, a common lot of frozen beefburgers from a producer in Poland was identified, lot A, distributed from December onward. A recall of lot A was initiated on 22 January 2015. No other French commercial or charitable groups received frozen beefburgers of the same lot from the Polish producer. No food samples of lot A from case homes were available for testing. However, international food trace-back investigations revealed that two samples from lot A tested positive in August 2014 for *Salmonella* spp. by the Polish producer in the context of controls requested by the specifications of the public contract with food bank A. After removal of the concerned part of the lot, a second series of samples in September 2014 tested negative and the lot was sent by the producer to France for distribution. For the other lots blocked by food bank A, no other elements (positive test results, human cases or information from Polish authorities) were known and they were put on the market with the authorisation of the DGAL.

Epidemiological investigations demonstrated that several cases identified after the second notification on 2 March 2015 began frequenting food bank A in February 2015, after the first recall, indicating that a second contaminated lot was in distribution. Frozen beefburgers from three different lots (B, C, D) were available for analysis in the homes of three cases. All of the available burgers were sampled two to three times. Analysis of 14 samples in six burgers from lot B yielded 12 positive results for *S.* Enteritidis. Seven samples from three burgers from lots C and D were negative.

All lots from the Polish producer were blocked as of 3 March 2015 and a recall of lot B was initiated on 6 March 2015. The rest of the lots remained blocked in France from distribution pending information from the Polish authorities regarding adherence to good hygiene practices (GHP) and Hazard Analysis Critical Control Point (HACCP)-based procedures by the Polish producer. This information was necessary to determine action regarding the remaining batch: further analyses, destruction, heat treatment, or return to the Polish producer.

Comparison by MLVA of 10 *Salmonella* strains identified in the human cases and 10 food samples showed the same profile 2_10_7_3_2 (VNTR loci order: SENTR7 SENTR5 SENTR6 SENTR4 SE3), confirming the link between cases from the entire outbreak period and with the contaminated frozen beefburgers from lot B (implicated in the second wave of the outbreak).

In both outbreak waves, the suspected lots of frozen beefburgers were distributed in several French departments in northern France; however, the majority of cases identified as having a link with food bank A were in the Somme department. Additional food trace-back investigations aimed to identify a potential explanation for the geographic distribution of cases (storage or distribution conditions at the local level, problems with the cold chain), but no such problems were identified. Analysis of NRC data did not identify any other French departments with an excess in cases of *S.* Enteritidis and no other region reported food-borne outbreaks citing food bank A or frozen beefburgers as the suspected source.

### European investigations

European health authorities were notified of outbreak investigations and the suspected link with frozen beefburgers from the Polish producer through a message on the EWRS on 10 March 2015. Messages to European epidemiologists and microbiologists were sent through the EPIS platform on 12 March 2015 sharing results of epidemiological and microbiological investigations. No other countries reported outbreaks of *S.* Enteritidis linked to consumption of frozen beefburgers from the Polish producer.

An alert was sent on the RASFF on 6 February 2015 (2015.0137) and a second one on 10 March 2015 (2015.0293) following the second outbreak. Through the RASFF, information obtained from Polish authorities did not indicate a failure to respect good hygiene practices by the Polish producer.

## Discussion and conclusion

Food-borne disease outbreaks due to *Salmonella* in France have been described in a variety of different food items including dried sausages, raw milk cheese and infant formula [8-11]. In 2010 an outbreak of *S.* Typhimurium linked to the consumption of beefburgers from Italy occurred in four schools in Poitiers, France, with over 550 confirmed cases [12]. While *S.* Enteritidis is typically associated with poultry and poultry products in France and elsewhere in the European Union [13,14] other food products may be the source of contamination. Most recently in 2014, a large outbreak of *S.* Enteritidis (displaying a different MLVA type) in the Hautes-Pyrénées department in the south of France was linked to the consumption of raw milk cheese with 181 confirmed and suspected cases identified (unpublished data). In the present outbreak, microbiological investigations confirmed the presence of *S.* Enteritidis in frozen beefburgers consumed by cases, corroborating the results of the epidemiological investigation.

The geographic distribution of cases primarily in the Somme department could be explained by several hypotheses. Regarding epidemiological investigations, several factors may contribute including (i) the population base for the department hospitals (a single large university hospital with paediatric emergency services in the Somme) may have centralised more cases than in other departments, leading to notification by the hospital; (ii) the occurrence of the outbreak in the winter during a period of increased cases of viral gastroenteritis which may have further decreased the likelihood that doctors prescribed stool analysis except in severe cases; and (iii) the exhaustiveness of food-borne outbreak surveillance in France.

Notably, the human *Salmonella* surveillance system is based on analysis at the NRC of *Salmonella* isolates sent on a voluntary basis by hospital and private laboratories, which is complemented by the mandatory notification of FBDOs. The exhaustiveness of these two complementary systems is estimated at 66% and 26% respectively [3,13]. While 11 FBDOs were identified among the cases, only one was notified to regional health authorities. The remaining 10 were identified through epidemiological investigations of confirmed cases notified by the hospital laboratory or the NRC. An excess of cases would likely have been detected by the weekly alert algorithm run at the NRC, but as the median delay from sample isolation to serotyping results is 14 days [15], the alert would have occurred several weeks into the outbreak. In this instance, the reactivity of hospital laboratory in notifying initial cases allowed for more timely epidemiological investigations and appropriate control measures.

Another possible explanation for the geographic distribution of cases, regarding food trace-back and microbiological investigations, is that the contamination was not homogenous, but concerned one or a few mixes (each lot was constituted of different mixes of ground beef processed over a given production period) in portions of the lots that were distributed primarily in the Somme department.

The described outbreak is unique in that it affected a specific population of individuals frequenting food bank A. A review of the literature regarding food-borne outbreaks did not return any articles regarding outbreaks linked to food distributed by food banks. Initial epidemiological investigations identifying a common link with food bank A for all families of confirmed cases was unusual and allowed for a more rapid orientation of epidemiological and food trace-back investigations after confirming the absence of other links between cases (commercial supermarkets, local markets).

This outbreak was also characterised by a large number of familial FBDOs identified during epidemiological investigations of confirmed cases, especially in the second wave. In the first outbreak wave, three FBDOs were identified in the families of 16 incident cases. In the second wave of the outbreak, eight families of 25 incident cases reported FBDOs. Furthermore, two-thirds of families reporting degree of doneness indicated that the burgers were consumed medium-rare or rare. Although it was not possible to quantify contamination levels, this could indicate that the frozen beefburgers were highly contaminated and that contamination levels may have been greater for the second lot recalled (lot B) based on the greater number of family FBDOs around incident cases.

The source of the outbreak was identified through epidemiological, microbiological and food trace-back investigations as frozen beefburgers originating from Poland. Large scale FBDOs requiring international trace-back investigation have been described for a wide variety of pathogens and food products [16-18]. Specifically, *Salmonella* outbreaks in European countries traced to imported food products have been previously reported, most recently *S.* Enteritidis linked to eggs from a German producer [19,20].

Such outbreaks can present difficulties related to the food trace-back investigations and control measures because numerous countries are implicated. This makes exchanges with different food safety authorities necessary to obtain information useful for the management of the outbreak such as information regarding hygiene practices by the producer, previous microbiological analyses in country of origin and return of remaining product. In this outbreak, the specificity of the food distribution chain for food banks presented challenges for food trace-back and management of remaining product. The frozen beefburgers were obtained through bids by producers for the Fund for European Aid to the Most Deprived (FEAD) and there is no commercial relationship between the food banks receiving products and the Polish producer. Analyses of the remaining lots based on a sampling plan established by the ANSES identified additional contaminated lots covering a production period of several months.

Overall, it is probable that the 44 cases with a confirmed link to food bank A in this outbreak represent just a small proportion of the actual number of cases that occurred in connection with the consumption of frozen beefburgers from food bank A. This outbreak highlights the important role of medical and laboratory personnel in the notification of unusual disease events that complements existing surveillance systems. The reporting of an unusual number of cases of salmonellosis in young children by a single hospital laboratory allowed for a rapid public health response that identified an unusual epidemiological link between the cases. Consequently, both national and international food trace-back investigations proceeded quickly, with timely information to European Union Member States that confirmed the absence of widespread distribution of the contaminated product, and led to a recall of contaminated lots and appropriate public health measures to ensure the safety of the remaining product.
